# Lithospheric mantle buoyancy: the role of tectonic convergence and mantle composition

**DOI:** 10.1038/s41598-019-54374-w

**Published:** 2019-11-29

**Authors:** K. Boonma, A. Kumar, D. Garcia-Castellanos, I. Jiménez-Munt, M. Fernández

**Affiliations:** 1 Institute of Earth Science Jaume Almera, ICTJA-CSIC, Lluís Solé i Sabaris, s/n, 08028 Barcelona, Spain; 2 Department of Earth and Ocean Dynamics, University of Barcelona, Barcelona, Spain

**Keywords:** Geophysics, Geodynamics, Geochemistry

## Abstract

Plate subduction and delamination, two key processes driving plate tectonics, are thought to be controlled by the buoyancy of the lithospheric mantle relative to the underlying asthenosphere. Most mantle delamination models consider a lithospheric density higher than the asthenosphere to ensure negative buoyancy (slab pull). However, mineral physics show that the continental lithospheric mantle density is lighter than the asthenosphere, and that only its pressure-temperature-composition dependence makes it become denser and unstable when sinking adiabatically. Here, we explore the controls on buoyancy using a 2D thermal-diffusive model of plate convergence, considering five chemical compositions and tectonothermal ages, namely Archon (>2.5 Ga), Proton (2.5–1.0 Ga), Tecton (<1.0 Ga), and two oceanic lithospheric plates of 30 Ma and 120 Ma. While the advection of colder rock in oceanic-like plates always results in negative buoyancy, Protons and Tectons exhibit an ability to slowly flip from negative to positive buoyancy at low convergence rates: they first favour the sinking due to advection and then become more buoyant because they are thinner and heat up faster during subduction. In contrast, the lighter density of cratons overprints this effect and hinders delamination or subduction, regardless of the convergence rate. This may explain why Archons are more stable during the Wilson cycle.

## Introduction

**Figure 1:**
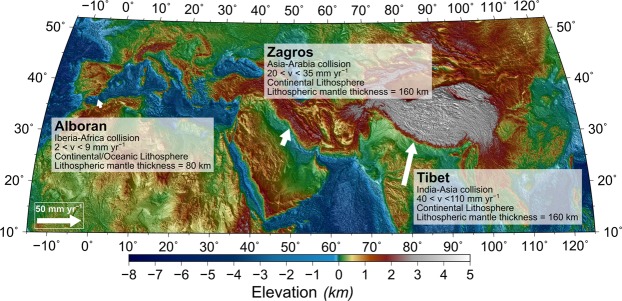
Geographic overview of regions with continental collision. Three regions within continental collisions that can be related to our study according to their convergence-rate and lithospheric mantle thickness.

Plate tectonics is thought to be mainly driven by the negative buoyancy of the lithospheric mantle relative to the asthenosphere, the driving force for both oceanic plate subduction and mantle delamination (the peeling off of the subcontinental lithospheric mantle from the crust and its sinking). The lithosphere interacts differently with the underlying asthenosphere in oceanic and continental domains. Oceanic lithosphere is formed at mid-ocean ridges and is soon (generally in less than 200 Ma) reworked back into the deeper mantle through subduction. In contrast, continental lithosphere is an order of magnitude older and has grown through accretion over longer time-scales, modifying its chemical composition. The continental lithosphere is believed to be recycled back into the asthenosphere at collision zones through the removal of parts of the lithospheric mantle by delaminating from the overlying crust. Geophysical and geological observations support mantle delamination in regions such as the Tibet^1,2^; Alboran domain^3–5^; the Apennines^6–8^; Eastern Anatolia^9–12^; and Sierra Nevada^13,14^, some of which are shown in Fig. 1. Numerical models suggest that this process is initiated by a conduit connecting the sublithospheric mantle with the weak lower crust and the negative buoyancy of the lithospheric mantle^15^.

Whereas most geodynamic studies have focused on the role of the viscosity contrast between the lower crust and the lithospheric mantle in the development of delamination^4,15–19^, or on the role of composition and thickness of the crust on the lithospheric subductability^20^, only few of them have scrutinized the influence of the density contrast between the lithospheric mantle and the asthenosphere^4,11,14,21–24^). These geodynamic models generally adopt densities that are either constant over time and space or temperature-dependent only, predefining a density for the subcontinental lithospheric mantle (SCLM) about 50 kg m^−3^ higher than of the asthenosphere. More recently, the role of pressure, temperature, and composition has been incorporated in calculating the buoyancy of the descending lithospheric mantle forced by plate convergence^24,25^.

Global geochemical analyses on mantle xenoliths, xenocrysts, and outcrops show that the mean composition of the SCLM is mainly related to the tectonothermal age of the overlying crust^26,27^. As the continental lithosphere undergoes cycles of melting, it gradually depletes in incompatible elements, such as Ca, Al, and Fe, relative to the primary source. The lithospheric mantle in Archean cratons (Archons), corresponding to tectonothermal ages >2.5 Ga, is generally highly depleted, while most lithospheric mantle beneath Neo-proterozoic/Phanerozoic mobile belts (Tectons, <1 Ga) is closer to the composition of the Primitive Upper Mantle (PUM). The SCLM beneath Meso- and Paleo-proterozoic shields and mobile belts (Protons), with tectonothermal ages of 2.5–1.0 Ga, is typically intermediate in composition. These compositional variations affect the bulk density of the SCLM and the greater the degree of depletion, the lower the density^27^. In contrast, the composition of the oceanic lithospheric mantle corresponds to that of PUM after melt extraction at mid-ocean ridges (MOR), being relatively homogeneous except beneath large oceanic plateaus.

Petrological and geochemical studies show that at identical P-T conditions as at the LAB, the density of the SCLM is lower than that of the PUM^27^ (see Supplementary Fig. S1), which is at odds with the aforementioned density distribution adopted in most geodynamic and static models^20^. In fact, the density of the sinking SCLM increases with pressure and decreases with temperature, thus P and T having competing effects on the depth-dependence of density. Whether mantle delamination or subduction are promoted by a negative buoyancy forced by plate convergence depends on the gradients of density relative to both parameters (Table 1).

**Table 1:** Physical parameters of all of the lithosphere types used in this study, together with those of the Primitive Upper Mantle (PUM).

Lithospheric Mantle type	Continental lithosphere T_Moho_ = 650 °C			Oceanic T_Moho_ = 300 °C		PUM
	Archon (Arc_3)	Proton (Pr_1)	Tecton (Tc_1)	120 Ma	30 Ma	
Lithospheric mantle thickness (km)	160	110	80	100	60	—
LAB depth (km)	200	150	120	110	70	—
${\Delta {\rm{\rho }}}_{{\rm{LAB}}}$ (kg m^−3^)	+68	+39	+19	+17	+17	—
Mg# (mantle fertility)	92.0	90.5	89.9	89.7	89.7	89.3
dρ/dP (kg m^−3^ MPa^−1^)	0.042	0.043	0.044	0.046	0.046	0.044
dρ/dT (kg m^−3^ K^−1^)	−0.1302	−0.1285	−0.1255	−0.1236	−0.1236	−0.1165
Density at 3GP/800 °C (kg m^−3^)	3319	3343	3365	3362	3362	3379
Density at 6GP/1300 °C (kg m^−3^)	3341	3367	3391	3389	3389	3407

Here we explore the idea that, whenever the lithosphere is incipiently forced to sink into the asthenosphere, it can become positively or negatively buoyant depending on its composition and on how the pressure-temperature evolution, imposed by plate convergence, affects its density evolution. This may explain why the older regions of the continental lithosphere (Archons) become tectonically more stable, self-prolonging their life span and favoring the Wilson cycle. To this purpose, we calculate the buoyancy of the sinking mantle in a kinematic model that accounts for a thermodynamically-consistent density dependence on temperature, pressure, and chemical composition.

## Method

**Figure 2:**
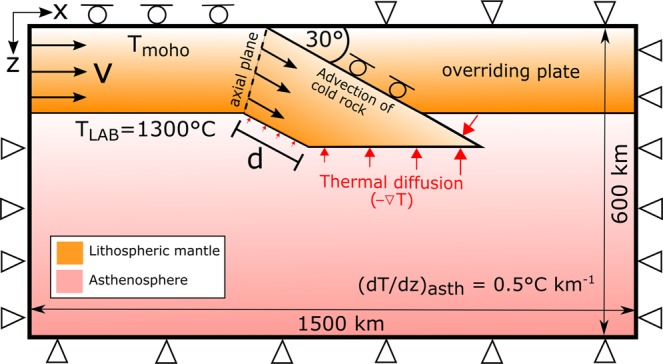
A conceptual model box. The model adopted for a lithosphere converging at a rate v over the underlying asthenosphere. The Moho depth is prescribed as 40 km for continental lithosphere and 10 km for oceanic lithosphere. The boundary conditions are: fixed temperature at the surface of the model (T_Moho_ = 650 °C for continental lithosphere and T_Moho_ = 300 °C for oceanic lithosphere) and at the lithosphere-asthenosphere boundary (LAB, T_LAB_ = 1300 °C); and no heat flow through the lateral sides of the model. The initial temperature distribution within the lithosphere is interpolated between the Moho and the LAB depth of each lithosphere type, while the adiabatic temperature gradient in the asthenosphere is fixed to $\mathrm{dT}/\mathrm{dz}=0.5\,^\circ {\rm{C}}\,{{\rm{km}}}^{-1}$. The thermal diffusivity of the lithosphere is set to 10^−6^ m^2^ s^−1^ while in the asthenosphere we use a higher value of 10^−5^ m^2^ s^−1^ to emulate mantle convection and adiabatic conditions. ‘d’ is the amount of shortening.

We developed a 2D kinematic approach to model plate convergence and subduction of the lithospheric mantle. The temperature distribution through time in absence of heat sources is given by

1 $$\frac{\partial {\rm{T}}}{\partial {\rm{t}}}=\nabla \cdot ({\rm{\alpha }}\nabla {\rm{T}})-\overrightarrow{{\bf{v}}}\nabla {\rm{T}}$$

where T is temperature (°C), t is time (s), α is thermal diffusivity (m^2^s^−1^), and $\overrightarrow{{\rm{v}}}$ is velocity (plate convergence rate, ms^−1^). The first term on the right side of Eq. (1) is related to thermal diffusion and the second term is related to thermal advection and Eq. (1) is solved with an explicit finite-difference scheme on a rectangular grid with a resolution of 5 × 5 km in a domain of 1500 × 600 km. Figure 2 shows the model setup and the related boundary conditions. As we focus on the density changes of the sinking lithospheric mantle, the model excludes the crustal layer.

The initial density distribution is calculated by considering a depth-dependent density according to

2 $$d{\rm{\rho }}=(\frac{\partial {\rm{\rho }}}{\partial {\rm{P}}}){\rm{dP}}+(\frac{\partial {\rm{\rho }}}{\partial {\rm{T}}}){\rm{dT}}$$

where the pressure $(\frac{\partial {\rm{\rho }}}{\partial {\rm{P}}})$ and temperature $(\frac{\partial {\rm{\rho }}}{\partial {\rm{T}}})$ derivatives for each lithosphere type are calculated by computing the stable mineral assemblages from major oxide compositions using Perple_X^28,29^ and averaging the obtained values of the derivatives over the lithospheric mantle thickness (Table 1). The considered lithospheric mantle types correspond to Arc_3 from the Slave craton, Pr_1 garnet-average, and Tc_1 garnet-average^27^, with their respective thicknesses to calculate representative density contrast at the LAB for Archean, Proterozoic, and Phanerozoic lithospheres, respectively (Table 1). The Moho temperature in continental lithospheres varies between 550 °C and 750 °C (see Supplementary Fig. S1), and in our kinematic model we assume a mean value of 650 °C. For oceanic lithosphere we assume a mean Moho temperature of 300 °C (see Supplementary Fig. S1).

The down-going lithospheric mantle is submitted to a prescribed velocity field, which is directed downward with an angle of 30°, parallel to the subduction plane (Fig. 2). Only the temperature of the lithospheric mantle is recalculated using Eq. (1). The temperature in the asthenosphere is fixed to the initial adiabatic gradient (0.5 °C km^−1^). In this way, we ensure that the asthenosphere does not cool down due to the heat transfer to the lithosphere, mimicking the effects of sublithospheric convection (for simplicity, it is not explicitly implemented in our model). Density variations are recalculated for each moving particle according to Eq. (2).

The buoyancy force at each time-step is calculated as the integral of the density changes relative to the initial stage over the entire subducting slab, *F*_*b*_ = *∫g*Δ*ρ dx dy*, where g is gravity and Δ*ρ* is the density difference between the slab and the asthenosphere. The buoyancy force, measured in N m^−1^, results from two components: diffusive (F_d_) and advective (F_a_), such that the total F_b_ = F_d_ + F_a_ is directed downwards when F_b_ < 0 (corresponding with slab pull).

## Results and Discussion

As lithospheric convergence initiates, the downward advection of cold lithospheric material generates a negative temperature anomaly that extends along the lithospheric slab, from the axial plane (Fig. 2) to a growing depth below the undisturbed LAB, depending on the convergence velocity. Simultaneously, thermal diffusion is responsible for the reduction of that negative temperature anomaly by transferring heat from the asthenosphere to the subducting slab. Therefore, heat advection and thermal diffusion have opposite effects on temperature anomaly and buoyancy. The prevalence of each depends on the convergence rate, and the composition/age of the lithospheric mantle and its thickness.

**Figure 3:**
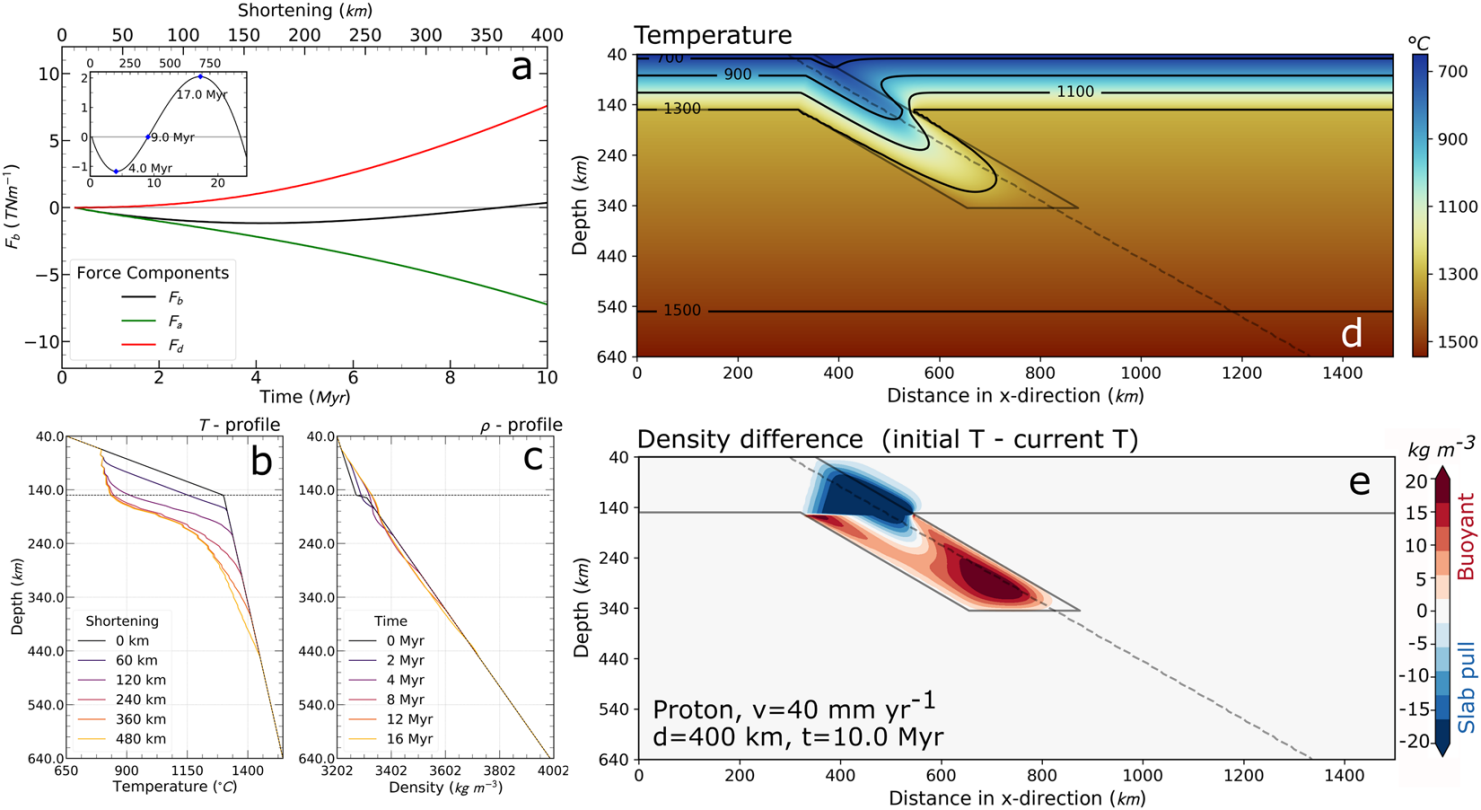
An example output of a model run. Proton lithosphere with v = 40 mm yr^−1^, at t = 10 Myr. (**a**) Evolution of the different components of Fb, due to advection (F_a_) and thermal diffusion (F_d_). The inset shows the whole evolution of Fb until the slab reaches the bottom of the model box. (**b**) Temperature profile along the slab (along the diagonal dashed line in (**d**) and (**e**)). (**c**) Density profile along the same line. The shortening legend in (**b**) corresponds to the time legend in (**c**). (**d**) Temperature distribution, with isotherm every 200 °C down to the LAB’s temperature of 1300 °C. (**e**) Density increase (blue) or decrease (red) relative to the initial density distribution. The integral of this density change across the subducting plate defines F_b_.

Figure 3a illustrates the contribution of advection and diffusion on the total buoyancy through time for an average Proton-type lithospheric mantle with a thickness of 110 km and a convergence rate of 40 mm yr^−1^. In this case (Fig. 3a inset), the buoyancy results negative from the beginning of the convergence up to 4.0 Myr of evolution, when it reaches a minimum value of −1.1 TN m^−1^ (downwards directed force) increasing to neutral buoyancy at 9 Myr. From here on, the buoyancy increases to a maximum of +2 TN m^−1^ at 17 Myr and decreases again. These time variations of the total buoyancy are related to the temperature and density variations occurring along the down-going slab (Fig. 3b,c).

### Effect of convergence rate and mantle composition

**Figure 4:**
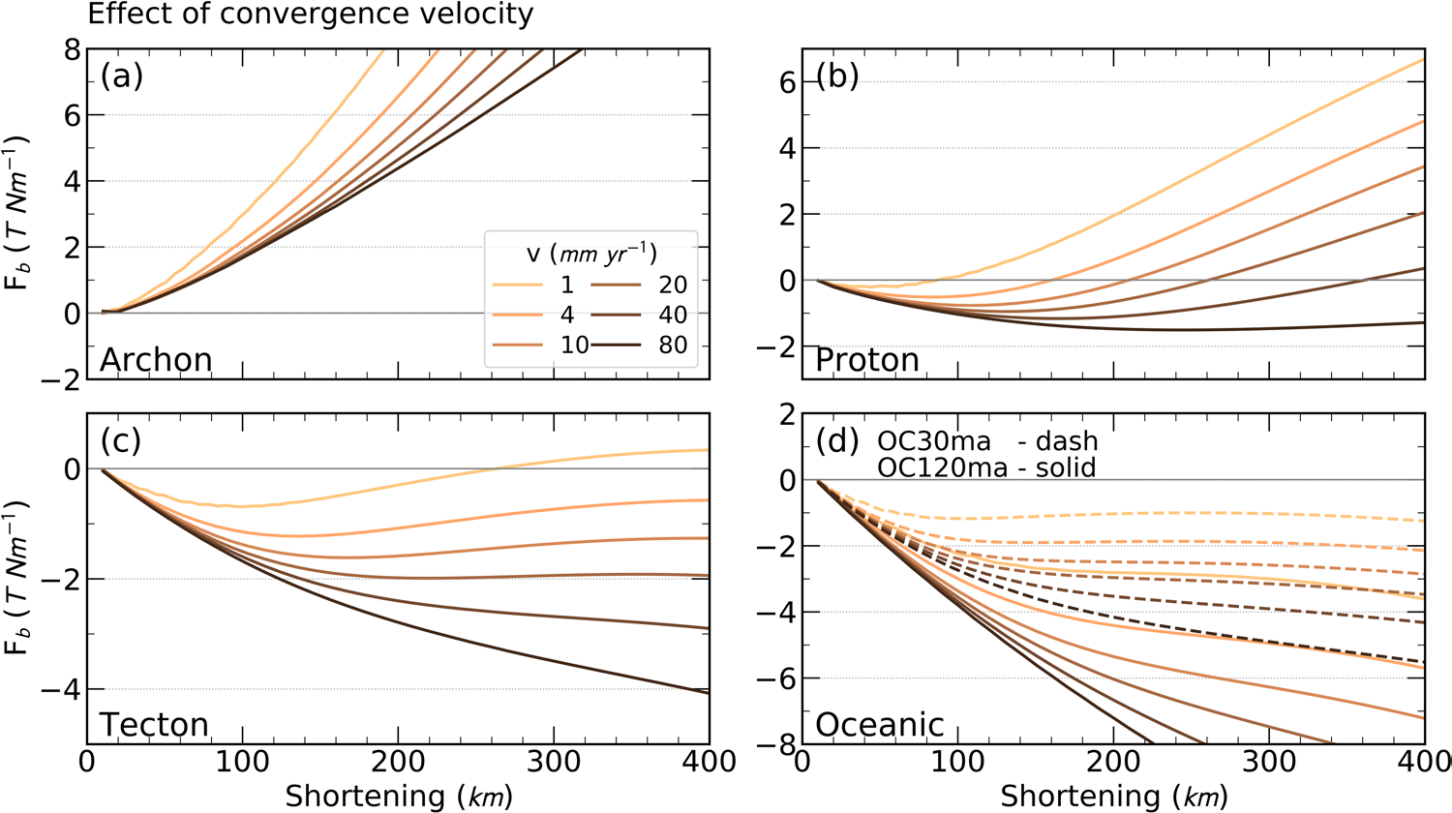
Effect of convergence rate on the total buoyancy force (F_b_). The five types of lithospheres are: (**a**) Archon, (**b**) Proton, (**c**) Tecton, and (**d**) Oceanic 30 (dash) and 120 million years old (solid) lithosphere.

The initial buoyancy of the lithospheric mantle is determined by the density contrast across the LAB $({\Delta {\rm{\rho }}}_{{\rm{LAB}}}={{\rm{\rho }}}_{{\rm{asth}}({\rm{LAB}})}-{{\rm{\rho }}}_{{\rm{lith}}({\rm{LAB}})})$, provided that P- and T- partial derivatives do not differ much for different compositions. Regardless of the convergence rate, an average Archon-like composition (160 km LM thickness) always shows positive buoyancy (F_b_ > 0) due to the large density contrast with the sublithospheric mantle at given P-T conditions with Δρ_LAB_ = +68 kg m^−3^ (Fig. 4a, Table 1). For an average Proton-like composition (Δρ_LAB_ = +39 kg m^−3^) with 110 km LM thickness, buoyancy initially has a negative trend (F_b_ < 0) for all convergence rates, after which F_b_ trends change and tending towards positive buoyancy after a time period spanning between 3 Myr for v = 80 mm yr^−1^, and 40 Myr for v = 1 mm yr^−1^(Fig. 4b). A younger average Tecton-like composition (Δρ_LAB_ = +19 kg m^−3^) and 80 km LM thickness shows trends with negative buoyancy of higher absolute values than those of Proton’s, F_b_ < 0 always (Fig. 4c). In the case of oceanic settings, both young (30 Myr) and old (120 Myr) lithospheres have monotonously decreasing buoyancies (F_b_ < 0) regardless of the convergence velocity (Fig. 4d), due to their intrinsically lower Moho temperature and lower density contrast across the LAB (Δρ_LAB_ = +17  kg m^−3^; Table 1).

The changing trend of the total buoyancy observed in Proton- and Tecton-type lithospheres is related to the low convergence rate, which allows for the subducting slab to thermally re-equilibrate with the surrounding asthenosphere, favouring thermal diffusion against advection and causing the slab to become more buoyant. On the contrary, fast convergence rates prevent the down-going slab from thermal re-equilibration because advection prevails on thermal diffusion. F_b_ becomes more negative (downwards slab pull) for higher convergence rates. The initiation of delamination or slab retreating should begin during this negative buoyancy stage, provided that the magnitude of the force and its duration suffice to trigger the process. The minimum (most negative) force for Protons occurs after 4 Myr (v = 40 mm yr^−1^) and 40 Myr (v = 1 mm yr^−1^) (Fig. 4b).

**Figure 5:**
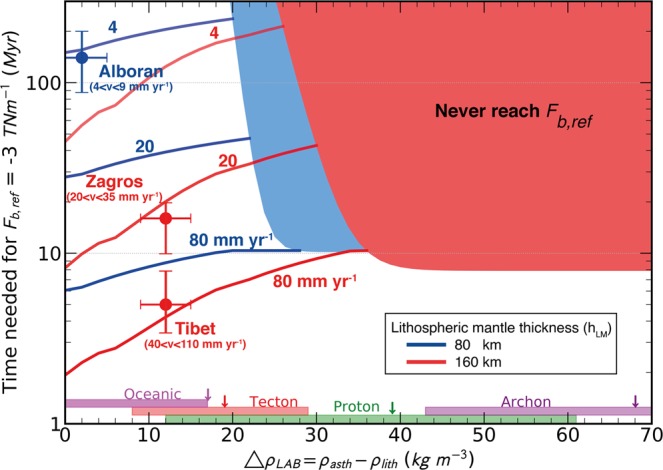
Combined effect of density contrast and convergence rate. Time needed to reach a downwards lithospheric buoyancy of F_b, ref_ = −3 TN m^−1^ as a function of the density contrast across the LAB for a velocity of plate convergence of 4, 20, and 80 mm yr^−1^. Curves correspond to a continent of lithospheric thickness of 80 (blue) and 160 km (red). The mantle type ranges at the bottom correspond to the various types of Tecton (8–29 kg m^−3^), Proton (12–61 kg m^−3^), and Archon (43–93 kg m^−3^)^27^. The density contrast for oceanic lithospheric mantle ranges from 0 to +17 kg m^−3^. The arrows indicate the density contrast values used for our model. The shaded areas indicate that the −3 TN m^−1^ value is not attained. Observations from three geological settings (Alboran^40^, Zagros^41^, and Tibet^41^) are shown. The horizontal error bars (±5 kg m^−3^) represent uncertainties in the average mantle composition; the vertical error bars correspond to the uncertainty in convergence rate.

We explore the time needed to attain a certain value for F_b_ that triggers delamination. We adopt a reference value of −3 TN m^−1^ which is a representative value for the plate tectonics driving forces^30–32^. Figure 5 displays the time needed to reach this value as a function of the density contrast across the LAB for two lithospheric thicknesses, 80 km and 160 km. The thicker lithospheric mantle takes less time to reach the reference pulling force (F_b_) and over a wider range of density contrast (Δρ_LAB_) than the thinner one. A faster convergence systematically leads to a wider compositional range for the triggering of delamination.

Finally, we isolate the effect of the lithospheric mantle thickness on the total buoyancy force, we vary this parameter while keeping a constant average Tecton-type composition with a convergence rate of 40 mm yr^−1^. The results in Supplementary Fig. S2 show that a thicker lithospheric mantle results in a more negative buoyancy, reaching the −3 TN m^−1^ first. As the thickness of the lithospheric mantle decreases the tendency for the positive buoyancy increases. The temperature and density distributions are similar for the three cases (Tecton-type), therefore, the diffusion of the slab occurs with a similar rate, leading to little difference in the buoyancy forces (see Supplementary Fig. S2). The advective components vary more since this component is due to the difference in how much material is being advected down i.e. thicker lithospheric mantle has a more negatively buoyant advective component.

### Tectonic relevance

The ability of the lithospheric mantle to delaminate or retreat is governed by its composition determining the density contrast relative to the underlying asthenosphere, and by the convergence rate. Our study shows that the oceanic lithosphere acquires negative buoyancy even at very low convergence rates and predicts ever-increasing slab pull during oceanic plate subduction, proportionally to the accumulated shortening and to the age of the plate. This is consistent with the observation that flat oceanic subduction is rare, requiring additional kinematic and rheological conditions to occur^33^. It is also consistent with the strong load (4 to 10 TN m^−1^) inferred from the flexural bending of the Pacific plate near the Tonga and Kermadec trenches, aged at 105 Ma^30^.

In contrast, the average Archon-type lithosphere always retains its positive buoyancy, whereas intermediate mantle compositions, corresponding to average Proton-type, can attain negative buoyancy depending on the convergence rate and the elapsed time. For convergence rates <70 mm yr^−1^, the maximum amplitude of negative buoyancy is attained after 80–250 km shortening (Fig. 4). This is similar to results from models of delamination of a lithospheric root during orogeny^16^, where it was concluded that lithospheric roots (equivalent to our ‘subducted slab’ portion) of at least 100–170 km length are needed to generate sufficient negative buoyancy for delamination and detachment to proceed.

The presented results are based on average chemical compositions representative of five lithospheric mantle types, and based on the assumption that convergence rates and compositions are constant through time. Mantle compositions and densities actually vary over a wide range even within given age and P-T conditions. The positive buoyancy of Archon’s is not warranted for lherzolitic mantle compositions, as in Kaapvaal (South Africa) and Almklovdalen (western Norway) cratons, showing densities 30 kg m^−3^ larger than average Archon-type and close to average garnet Proton-type^27^. Similarly, Proton’s with harzburgite compositions can have densities 32 kg m^−3^ lower than the average Proton-type and will never get negative buoyancy. Moreover, metasomatic processes can change the major-element mantle composition converting harzburgite/dunite (lighter end-member) to lherzolite (heavier end-member) and back, modifying its buoyancy^34^. Convergence rates may also vary through time, especially for slow speeds (v ≤ 20 mm yr^−1^) since they involve long lasting processes (20–150 Myr) to attain significant shortening. These buoyancy variations, caused either by lateral compositional heterogeneities, metasomatic and differentiation processes, far-field tectonic stresses, or a combination of these, can contribute to isostatic destabilization of cratons by dripping and detachment of heavier mantle portions, as can be the case of western Gondwana craton^35^.

**Figure 6:**
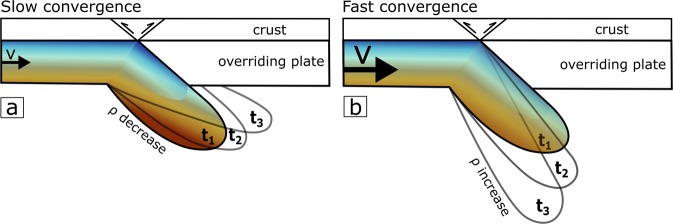
Cartoon summarizing the effect of the convergence rate. (**a**) shows the case with a slow convergence rate which allows for great thermal diffusion, which in turn, reducing the slab’s density and the slab becomes more buoyant. (**b**) shows the case with a faster convergence rate, which allows the slab to keep cooler and denser, leading to a higher density and an increase in negative buoyancy.

An outstanding outcome of the model is the predicted change from negative to positive buoyancy through time for continental subduction. The initial density contrast across the LAB determines how soon diffusion overcomes advection such that intermediate density contrasts 20 kg m^−3^ ≤ $\Delta {{\rm{\rho }}}_{{\rm{LAB}}}$ ≤ 50 kg m^−3^ and convergence velocities ≤50 mm yr^−1^ eventually lead to a change from negative to positive buoyancy (Fig. 4). This may promote a rising of the subducting continental lithosphere and a subsequent flattening below the overriding plate producing lithosphere underthrusting as proposed in the India-Eurasia collision region (Fig. 1 and Fig. 6). According to seismic tomography and potential field modelling^36,37^, underthrusting beneath the western Himalaya-Tibet extends 200 km beyond the suture, whereas to the east, the convergence is accommodated by a steep subducting slab. This two-fold behaviour is compatible with a 160-km-thick lithospheric mantle of Proton-type and subducting at a high velocity of 45 mm yr^−1^ (Fig. 5). Clearly, our model disregards other relevant mechanisms such as the weight of oceanic lithospheric slabs subducted before collision or the role of subduction of the continental crust, and hence quantitative comparisons with real scenarios must be taken with caution^19,20,24,25,38,39^.

Figure 5 also shows three geological scenarios (Alboran^40^, Zagros^41^, and Tibet^41^, located in Fig. 1) that can be quantitatively compared to our study. The Alboran Basin is a back-arc setting characterised by a thinner lithospheric mantle^42^ (80 km) undergoing Cenozoic mantle subduction, and possibly late Miocene delamination^43^, whereas Zagros and Tibet are in the thicker lithospheric mantle group^37^ (red lines from Fig. 5, 160 km). The three regions are clustered on the left side of the plot (low ${\Delta {\rm{\rho }}}_{{\rm{LAB}}}$). Our model suggests that in order to develop negative buoyancy under higher ${\Delta {\rm{\rho }}}_{{\rm{LAB}}}$ as in cratons, the convergence rate should be higher than 80 mm yr^−1^ or remain forced for tens of million years, making it a rare phenomenon (Fig. 6).

In summary, the results show that incorporating realistic mineralogy-based densities to geodynamic models rises up an unforeseen control on the development of negative buoyancy. Whereas assigning a constant or temperature-dependent higher density to the lithosphere always results in slab pull, accounting for the effects of composition and pressure reveals that the plate convergence velocity is key to determine the development of negative buoyancy, delamination, and subduction.

The model designed here provides a methodological framework for understanding the stability of the lithosphere during the convergence of tectonic plates, and suggests a simple thermodynamic explanation for the long-term preservation of older continental regions (cratons) in the Wilson cycle. As continents aggregated during the early Earth evolution, their average buoyancy relative to the asthenosphere increased, making them less prone to subduct or delaminate and hence more stable. However, even Archean and Proterozoic lithospheric plates retain a chance to become recycled into the mantle if they are forced to sink by a fast-enough plate convergence, depending on the geographical configuration of tectonic plates.

## Supplementary information

Supplementary Information
