# Author Correction: Lithospheric mantle buoyancy: the role of tectonic convergence and mantle composition

**DOI:** 10.1038/s41598-020-57833-x

**Published:** 2020-01-15

**Authors:** K. Boonma, A. Kumar, D. Garcia-Castellanos, I. Jiménez-Munt, M. Fernández

**Affiliations:** 1 Institute of Earth Science Jaume Almera, ICTJA-CSIC, Lluís Solé i Sabaris, s/n, 08028 Barcelona, Spain; 2 Department of Earth and Ocean Dynamics, University of Barcelona, Barcelona, Spain

Correction to: *Scientific Reports* 10.1038/s41598-019-54374-w, published online 29 November 2019

The Acknowledgements section in this Article is incomplete.

“This work has been supported by EU Marie Curie Initial Training Network ‘SUBITOP’ (674899-SUBITOP-H2020-MSCA-ITN-2015) and MITE (Spanish Government national research program; CGL2014-59516). We thank the anonymous reviewers whose comments helped fine-tuning the manuscript.”

should read:

“This work has been supported by EU Marie Curie Initial Training Network ‘SUBITOP’ (674899-SUBITOP-H2020-MSCA-ITN-2015) and MITE (Spanish Government national research program; CGL2014- 59516). The Laboratory of Geodynamics at ICTJA-CSIC provided the computing facilities. We thank the anonymous reviewers whose comments helped fine-tuning the manuscript.”

Additionally, the Author Contributions section in this Article is incorrect.

“K. Boonma wrote the main manuscript text, built and ran the numerical modelling code, and prepared all of the figures. A. Kumar calculated and provided the mineralogical parameters, as well as assisted with the numerical modelling code (available at https://github.com/kboonma/LithBuoy). M. Fernández and D. Castellanos assisted with the major rewrites. All authors reviewed and edited the manuscript. M. Fernández, D. Castellanos, and I. Jiménez-Munt jointly supervised the work.”

should read:

“K. Boonma wrote the main manuscript text, built and ran the numerical modelling code (available at https://github.com/kboonma/LithBuoy), and prepared all of the figures. A. Kumar calculated and provided the mineralogical parameters, as well as assisted with the numerical modelling code. M. Fernández and D. Garcia-Castellanos designed the study. All authors reviewed and edited the manuscript. M. Fernández, D. Garcia-Castellanos, and I. Jiménez-Munt jointly supervised the work.”

