# Supplementary material for: Lithospheric mantle buoyancy: the role of tectonic convergence and mantle composition
Source: Sci Rep. 2019 Nov 29;9:17953. doi: 10.1038/s41598-019-54374-w (PMC6884500; doi:10.1038/s41598-019-54374-w)
Supplement: Supplementary file 1 — Supplementary Information [file 41598_2019_54374_MOESM1_ESM.docx]

**Lithospheric mantle buoyancy: the role of tectonic convergence and mantle composition**

K. Boonma^1,2 *^, A. Kumar^1,2^, D. Garcia−Castellanos^1^, I. Jiménez−Munt^1^, M. Fernández^1^

^1^Institute of Earth Science Jaume Almera, ICTJA−CSIC, Lluís Solé i Sabaris, s/n, 08028 Barcelona, Spain

^2^Departament de Dinàmica de la Terra i de l'Oceà, Universitat de Barcelona, Barcelona, Spain

Corresponding author: Kittiphon Boonma ([kittiphon.b@gmail.com](mailto:kittiphon.b@gmail.com))

# Supplementary Materials

Supplementary Table S1. Thermophysical properties used for the calculation of temperature and density in Supplementary Figure S1.

| **Crust** | **Body** | **Density**  **(kg m^−3^)** | **Thermal Conductivity**  **(W m ^−1^ K ^−1^)** | **Radiogenic Heat Production**  **(W m^−3^)** |
| --- | --- | --- | --- | --- |
| Oceanic | Sediments | 2350 | 2.5 | 1.0E−06 |
|  | Oceanic Crust | 2950 | 2.1 | 0.3E−06 |
| Continental | Upper Crust | 2750 | 2.5 | 1.0E−06 |
|  | Lower Crust | 2950 | 2.1 | 0.3E−06 |

#
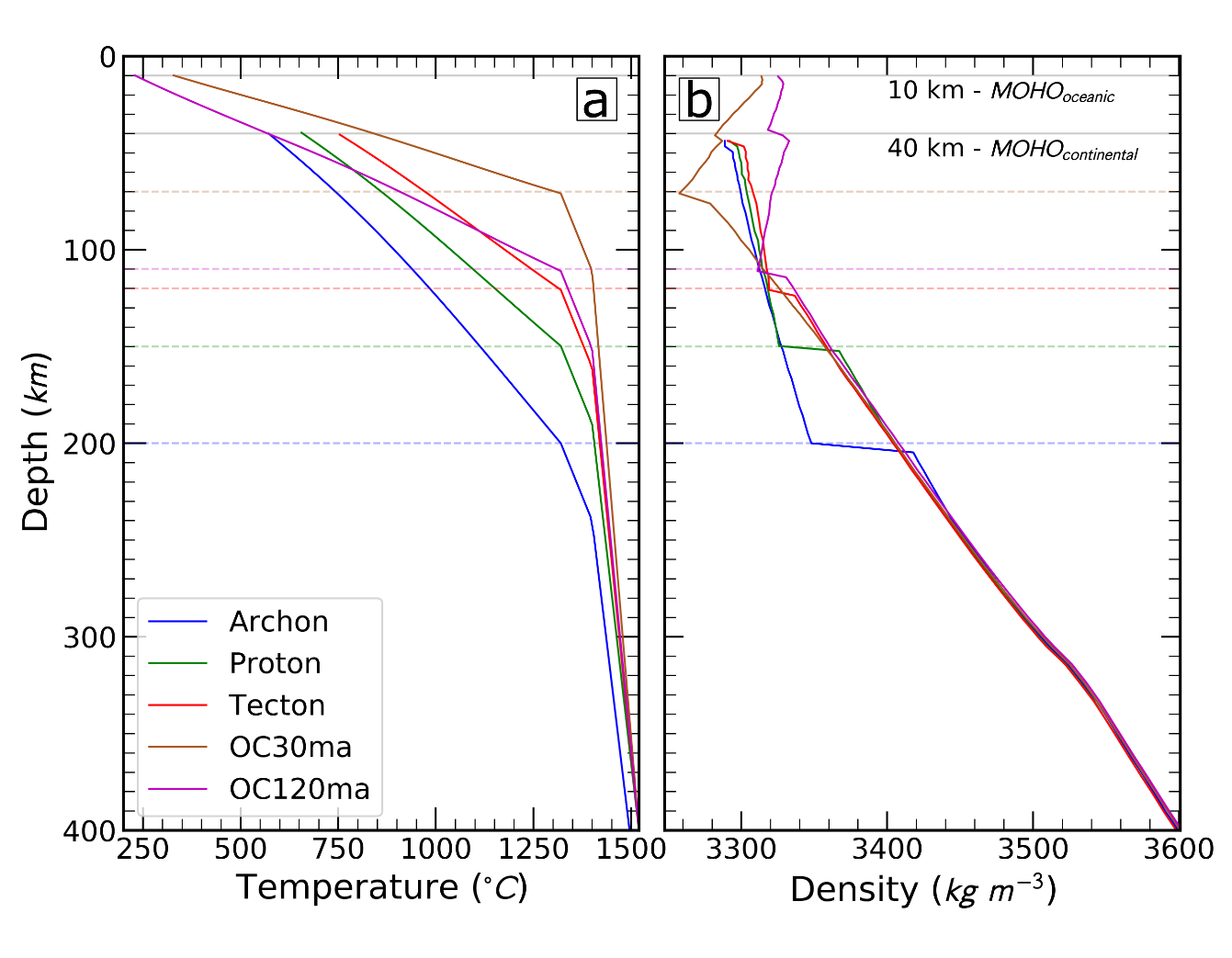


Supplementary Figure S1 **Temperature (a) and density (b) profiles for the five lithosphere types considered**. The temperature is calculated by solving for the steady−state heat flow equation with conductive regime in the lithosphere and adiabatic gradient in the asthenosphere. The physical properties used for the crust are listed in Supplementary Table S1. Thermal conductivity and density in the mantle are calculated by solving for stable mineral assemblages from major oxides compositions using Perple_X. It should be noted that these profiles are not the initial profiles used in the models, but they are the reference profiles.


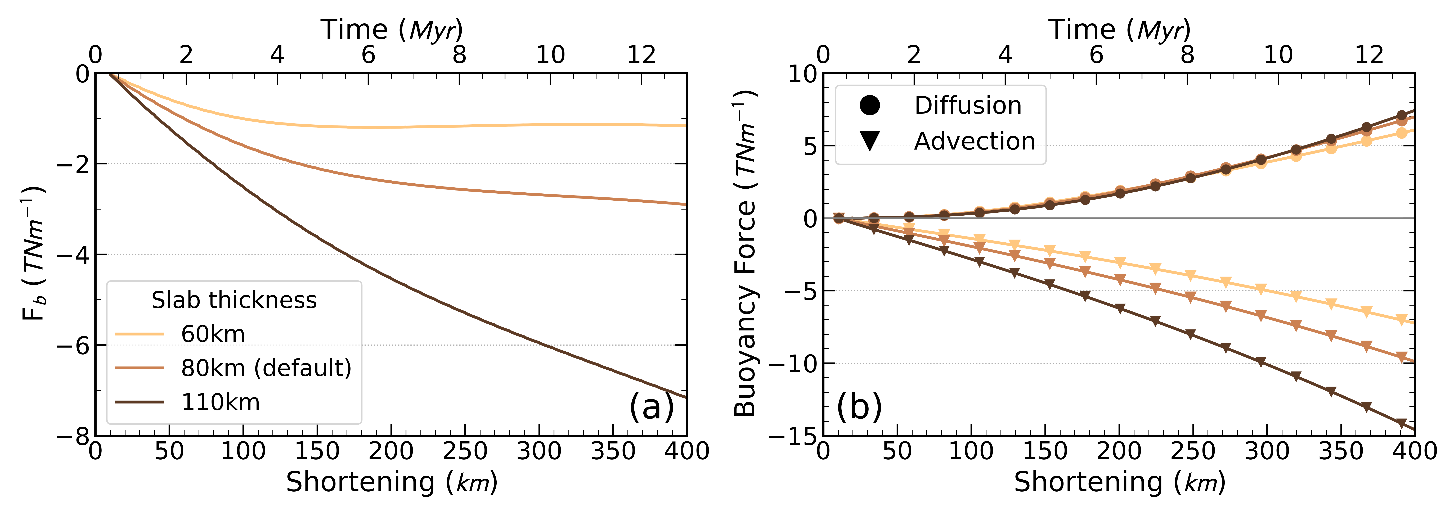


Supplementary Figure S2 **Effect of lithospheric thickness on Tecton lithosphere**, at convergence rate v = 40 mm yr^−1^. (a) Total buoyancy force (F_b_) for each thickness: 60 km (LAB 100 km); 80 km (LAB 120 km); and 110 km (LAB 150 km). (b) The advective (F_a_) and diffusive (F_d_) components contributing to the corresponding F_b_ in (a). This figure shows that diffusive and advective components of the buoyancy force have similar trends for all lithospheric thicknesses though showing that the thicker the lithosphere the larger the amplitudes of the corresponding components. Despite these similar trends in the components, the total buoyancy force differs noticeably with the lithosphere thickness in both the maximum of negative buoyancy force and the stage it occurs. A thicker lithospheric mantle results in a more negative buoyancy.
